# The Overgrowth of Surgery in Gynæcology

**Published:** 1886-05

**Authors:** 


### The Overgrowth of Surgery in Gynæcology.

Under this title we notice an extract in the *Obstetric Gazette*, the contents of which cannot be better reproduced than by quoting it verbatim: "A. J. C. S., in the *Medical Digest*, judging from current literature, one gets the impression that the whole art of gynæcology is reduced to abdominal surgery, with an occasional plastic operation about the cervix uteri or perinæum. The published transactions of the gynæcological and obstetrical societies of the larger cities of this country are made up of great operations. The removal of the ovaries, tubes, uterus, and all forms of tumors connected with these organs, seems to be the whole occupation of those who practice gynæcology. If these specialists and their assistants condescend to treat the diseases of women which do not require heroic surgical treatment, they evidently do not consider such practice worth mentioning. There is certainly great surgical activity in this department, so much so, that we are led to hope that a reaction may soon come — a change which will bring up the medical side of the subject. It is time that the knowledge, judgment and skill of the physician should receive as much attention and consideration as the daring operations of the surgeon. In the crop of gynæcologists coming up at this time, we find nearly all of them thirsting for big operations like hysterectomy and ovariotomy. It would be well if these were men who could be called physicians.

